# A Roadmap to the Brittle Bones of Cystic Fibrosis

**DOI:** 10.4061/2011/926045

**Published:** 2010-12-16

**Authors:** Ashwini P. Gore, Soon Ho Kwon, Antine E. Stenbit

**Affiliations:** ^1^Division of Endocrinology, Diabetes & Medical Genetics, Department of Medicine, Medical University of South Carolina, Charleston, SC 29425-6300, USA; ^2^Division of Pulmonary, Critical Care, Allergy & Sleep Medicine, Department of Medicine, Medical University of South Carolina, Charleston, SC 29425-6300, USA

## Abstract

Cystic fibrosis (CF) is an autosomal recessive disorder which despite advances in medical care continues to be a life-limiting and often fatal disease. With increase in life expectancy of the CF population, bone disease has emerged as a common complication. Unlike the osteoporosis seen in postmenopausal population, bone disease in CF begins at a young age and is associated with significant morbidity due to fractures, kyphosis, increased pain, and decreased lung function. The maintenance of bone health is essential for the CF population during their lives to prevent pain and fractures but also as they approach lung transplantation since severe bone disease can lead to exclusion from lung transplantation. Early recognition, prevention, and treatment are key to maintaining optimal bone health in CF patients and often require a multidisciplinary approach. This article will review the pathophysiology, current clinical practice guidelines, and potential future therapies for treating CF-related bone disease.

## 1. Introduction

Cystic fibrosis (CF) is an autosomal recessive disorder caused by defects in the cystic fibrosis transmembrane conductance regulator protein (CFTR), a chloride channel found in the epithelial tissues in the lungs, sinuses, pancreas, skin, and gastrointestinal tract. CF most commonly affects Caucasians and occurs with a frequency of 1 in 2000 to 3000 live births in the United States each year [1]. The defects in CFTR leads to alterations in the sodium, chloride, and water transport in the epithelial cells and in turn to changes in the viscosity and hydration of the fluids overlying the epithelial cells. The change in the fluid composition is partially responsible for several of the complications associated with the progression of CF such as chronic respiratory infections, pancreatic duct obstruction, pancreatic insufficiency, biliary obstruction, cirrhosis as well as distal intestinal obstruction syndrome. In addition to expression of CFTR in a variety of epithelial cells, its expression has been found in osteoblasts but its precise role in these cells remains to be elucidated [2, 3]. The respiratory disease is hallmarked by bronchiectasis caused by cycles of infection, inflammation, and destruction of the airways. Airway clearance and aerosolized therapies have been a staple in the care of the CF patients for the past several decades and have lead to an improvement in the lifespan of the patients leading some to consider it now a life-limiting instead of a fatal disease. However, CF continues to lead to premature respiratory failure from repeated exacerbations, chronic infection by *Pseudomonas aeruginosa*, *Staphylococcus aureus*, *Burkholderia cepacia*, or other pathogenic organisms. 

The rapid advancements in medical therapy and patient care discussed above have increased the median predicted survival age for patients with CF to 37.4 years (http://www.cff.org/). In the coming years, the number of patients with CF who are over the age of 18 will surpass those who are in the pediatric age group; currently approximately forty-six percent of the CF population in the United States is over 18 years of age. As a consequence, the various disease-related complications that were only seen in a sub-set of the CF population are now being seen in a larger number of patients, such as osteoporosis and osteopenia. The secondary bone loss seen in the CF population is multifactorial, however can cause significant morbidity in the adult population.

## 2. Bone Disease in CF

Bone disease in patients with cystic fibrosis was first described over 3 decades ago and is characterized by decreased mineral density, increased fracture rates, and kyphosis [4, 5]. Unlike bone loss seen in postmenopausal women, bone loss in the CF population begins at a young age and continues as the patient ages. The prevalence of bone disease in the CF population increases with age and has been correlated with severity of lung disease [6–11]. However, one is more likely to see an adult CF patient with low bone mineral density than with normal bone mineral density even with normal lung function. 

A recent meta-analysis by Paccou et al. reported that the prevalence of osteoporosis and osteopenia in young adults with CF was 23.5% and 38%, respectively [12]. Multiple cross-sectional studies have demonstrated an increased incidence of fractures in individuals with CF with vertebral fractures being the most common followed by rib fractures [4, 5, 13–15]. Another common skeletal problem seen in the CF population seen as early as the third decade of life is kyphosis [13, 16, 17]. Some studies have noted it to be between 10%–40% CF patients [16, 17]. The development of bone disease in the CF population can lead to significant issues with loss of lung function, deformities, and also increased pain issues. One can imagine that a vertebral or rib fracture would make it difficult to maintain a regimen of airway clearance that is needed for the prevention of CF exacerbations and the maintenance of lung function.

Although most of the pathologic consequences occur during adulthood in the CF population, there have been several studies that indicate that during childhood and puberty the CF patients achieve approximately half of the bone density than their healthy counterparts [18–22]. Puberty is especially important for the development of bone density and is a time where there is both peak growth velocity and bone density accrual. The reduction in the bone formation in the CF patients may be due to a combination of their delay in puberty, chronic infections, and hormonal imbalances. Numerous cross-sectional and longitudinal studies suggest that children and adolescents with cystic fibrosis fail to achieve adequate bone mass as compared to healthy controls during their pubertal growth spurt [18–20, 22]. To compound this decrease in bone density in puberty and adolescence, clinical studies show annualized rates of loss in BMD in the CF patients approach those seen in postmenopausal women [23–25]. Lower baseline bone density coupled with increased losses puts CF individuals at higher risk of low BMD.

The maintenance of bone health is also essential for the CF population as they approach the time for lung transplantation. Severe bone disease can lead to exclusion from lung transplantation, which is often a life-saving treatment for many CF patients. The most effective strategies have been found to be early recognition, prevention, and treatment in this population. This article will discuss the pathogenesis, the current guidelines, and potential therapies for the CF population.

## 3. Pathogenesis

### 3.1. Histologically

Three of the studies that have looked at bone histomorphometric data in CF adults with low BMD have shown a decrease in cortical as well as trabecular bone volume and a decrease in connectivity [26–28]. At a cellular level, there is a disruption in the delicate balance between osteoblast and osteoclast activities. The osteoblastic activity is decreased in the bones of the CF patients due to decreased osteoblast number and their biosynthetic potential that is compounded by osteoclast activity which is increased, primarily through increased osteoclast number. The rise in bone resorption when compared to new bone formation leads to low bone density in this population even in patients who are clinically stable [26, 28]. The exact cause of the disruption in this balance has not been completely been elucidated; however, there are studies to suggest that mutations in CFTR itself may play a role in a portion of the CF patients. Interestingly, even though vitamin D deficiency is very common in individuals with CF and is an important etiologic factor for the low BMD observed in this condition, osteomalacia is usually not a feature of CF-related bone disease [28].

## 4. Possible Role of CFTR

There are three observations that indicate that CFTR has a direct association with the loss in bone density in the CF patients. First, CFTR has been shown to be expressed in human osteoblasts, osteocytes, and osteoclasts [3]. Second, CF patients with at least one ΔF508 allele had significantly lower Z-scores than those with other genotypes, which suggests a direct association between CF-related bone disease and the ΔF508 mutation [29]. Finally, experiments in CFTR-deficient mice demonstrated a decreased BMD with more cortical bone thinning and altered trabecular architecture as compared to control mice despite lacking other overt manifestations of CF (e.g., lung disease and pancreatic insufficiency) [30]. Together these findings suggest that loss of CFTR function may adversely affect bone density. However, the exact role of CFTR, specifically the ΔF508 allele in CF-related bone disease, is yet unclear.

In addition, the loss or reduction in CFTR activity has been shown to lead to chronic inflammation through a decrease in osteoprotegerin and a concomitant increase in prostaglandin E2 [2]. The alteration of osteoprotegerin and prostaglandin E2 increases the inflammation-driven bone resorption [2]. This would further indicate that there is a direct association between CFTR and the BMD in the CF patients. These studies have increased the interest in the possible role of CFTR gene in bone development and pathophysiological processes. Once again however, it is not known whether CFTR has a direct or indirect effect on bone formation.

## 5. Factors Influencing Bone Loss

The development of bone disease in the CF patients is thought also to be secondary to their chronic illness and is influenced by multiple factors. It is essential to remember that nutrition, lung disease, and bone health are all related in this population. (Table 1).

## 6. Malnutrition and Pancreatic Insufficiency

Optimum nutrition is vital not only for pulmonary health but also for bone health [17, 31–33]. Malnutrition seen in the adult CF patients is contributed by pancreatic insufficiency, increased catabolism due to chronic infections, and body images issues which afflicts many of the CF patients. A large portion of CF patients are pancreatic insufficient which leads to inadequate secretion of pancreatic enzymes and malabsorption of fat soluble vitamins (A, D, E, and K), calcium, and other macronutrients which play a key role in bone formation [34]. Despite pancreatic enzyme replacement therapy, the majority of CF patients have body mass index that are in the low normal or malnutrition range. A great deal of energy is spent on dietary education and nutritional support in an attempt to improve the body mass index of the CF population. Low body mass index has been linked to low bone mineral density, especially in the adolescent and young adults [35, 36]. 

### 6.1. Vitamin Deficiencies

One vitamin deficiency which is prevalent in the CF population is Vitamin D deficiency, regardless of season or latitude [37–40]. In addition to pancreatic insufficiency and malabsorption described above, multiple other factors such as inactivity and decreased sunlight exposure, low body fat (reduced Vitamin D stores), reduced 25 hydroxylation, increased metabolic degradation, and decreased vitamin D binding protein have all been implicated as potential etiologies for the low vitamin D reserves. 

In non-CF individuals, Vitamin D deficiency in children results in failure to achieve optimal height and peak bone mass or even overt rickets while in adults it is associated with impaired bone density due to decreased calcium absorption and hyperparathyroidism as well as osteomalacia. Although Vitamin D deficiency is commonly seen in CF population, osteomalacia in not a feature of CF-related bone disease, suggesting that other etiologies play an equally important role in pathogenesis [28].

Even with replacement therapy, vitamin K is known to be depleted in up to 40% of the CF patients. The significance of vitamin K to bone health has been linked to osteocalcin. A depletion of vitamin K has been associated with higher levels of undercarboxylated osteocalcin. Several studies have looked beneficial role of vitamin K supplementation specifically on the increased carboxylation of osteocalcin and improvement in bone formation [38, 41–44].

## 7. Cystic Fibrosis-Related Diabetes

Patients with CF can develop impaired glucose tolerance (IGT) or even frank diabetes due to the progressive pancreatic damage caused by defective acinar and ductular secretions. This type of pancreatic damage may not be similar to classic Type I or Type II diabetes; however, the resultant abnormalities in glucose and insulin regulation can affect bone health [45–47]. Just as in patients without CF, cystic fibrosis-related diabetes and IGT may play an important role in the development of bone loss [45].

## 8. Gonadal Steroid Deficiency

Puberty is a crucial period in which peak bone mass accrual occurs. CF individuals are frequently noted to have delayed puberty and decreased overall bone mineral accretion. Adolescents with CF have lower sex hormone levels as compared to healthy age matched controls, but most have normal values when adjusted for Tanner stage [48, 49]. Hypogonadism can cause accelerated bone loss. The lower peak bone mass combined with increased bone losses may contribute to bone disease, but observational studies have not observed a consistent association [48, 49].

## 9. Physical Inactivity

Just as with some of the general population, the CF population is not as physically active as they should be. Although there are limitations with many of the patients with severe lung disease and there is social stigmata attached to the chronic cough that the CF patients have especially when exercising, any reduction in activity or weight bearing activity in this population has negative impact on their BMD. The CF patients are often burdened with a significant treatment burden and in the adult population frequent pulmonary or sinus exacerbation. For all of these reasons, there is a decrease in total activity hours and weight-bearing activities in this population even in light of the fact that it has been shown not only to improve bone health but also pulmonary health [32, 50–53].

## 10. Recurrent Infections

As stated previously, there is an association between the severity of lung disease and CF-related bone disease [5–10]. During acute exacerbations of lung disease, markers of bone turnover tend to rise and their levels drop after appropriate treatment with antibiotics, chest physical therapy, improved nutrition, and other supportive measures [54]. Systemic hormones, inflammatory cytokines, and localized growth factors can all affect bone remodeling [55]. Elevated levels of cytokines and growth factors like tumor necrosis factor-*α* (TNF-*α*), vascular endothelial growth factor (VEGF) and IL-1, IL-6 and IL-11 found in the serum and respiratory tract of CF patients may stimulate osteoclast-mediated bone resorption and inhibit bone formation [56].

## 11. Glucocorticoids

It is well established that glucocorticoids negatively impact bone health. Their primary effect on bone is to suppress formation of osteoblasts and promote apoptosis of osteoclasts and osteocytes. Systemic glucocorticoids can cause a rapid bone loss within the first few months of treatment, followed by a slower 2%–5% loss per year with chronic use [57]. CF patients with symptoms and signs of asthma or allergic bronchopulmonary aspergillosis are often treated with systemic or oral steroids to improve lung function. In most studies, the dose and duration of systemic steroid use impacted the decreased bone mineral density [58–62]. The use of inhaled corticosteroids, although associated with short term changes in markers of bone turnover, has not been shown to affect BMD or fracture risk, independent of severity of underlying lung disease [58, 62].

## 12. Lung Transplantation and Immunosuppressive Therapy

Lung transplantation may be potentially lifesaving in CF patients with end stage lung disease. Patients awaiting lung transplants almost always have a low BMD due to factors discussed above [63, 64]. Immediately following the transplant, a rapid decline in BMD is noted and may be attributed to limited mobility, chronic steroid use, and use of other immunosuppressive agents [65–70]. This decreased BMD is associated with an increased fracture risk. Though BMD may stabilize or even increase a year or more after transplant, the fracture risk appears unchanged [65].

## 13. Guidelines to Improve Bone Health in CF

The clinical practice guidelines were outlined by the CF foundation committee for optimizing bone health in CF patients in The Journal of Clinical Endocrinology & Metabolism in 2005 and are scheduled to be revised within the next year [13]. 

### 13.1. Screening for Bone Disease

The CF foundation bone health committee recommended that all adults with CF should have a baseline BMD at age 18 years. Children age 8 years or greater with other risk factors (i.e., ideal body weight <90%, FEV1 <50% predicted, glucocorticoids 5 mg/day or more for 90 days/year or longer, delayed puberty, or previous history of fractures) should also be screened.

The committee cautioned that while interpreting DEXA scan results, Z-scores should be used up to age 18, Z- or T-scores are nearly equivalent between ages 18 and 30, while T-scores should be used above the age of 30. The recommendations are summarized in Table 2.

### 13.2. Preventive Strategies

The preservation of optimal bone health in CF requires a multisystemic approach. Good nutrition is critical in maintaining lean body mass and overall bone health. Vitamin and macronutrient deficiencies should be adequately repleted, and a BMI >50% percentile should be targeted. Pulmonary infections should be treated aggressively, and every effort should be made to maintain adequate glycemic control and to minimize the use of steroids whenever possible. Weight bearing exercises are well known to increase BMD in healthy subjects and are highly recommended in preventing bone loss in CF individuals.

## 14. Therapeutic Considerations

### 14.1. Vitamin D Supplementation

As discussed previously, Vitamin D insufficiency is common among CF patients and is associated with bone disease. An optimum level has not been established but based on observations in non-CF patients that parathyroid hormone levels start to increase when 25OH Vit D levels fall below 30 ng/mL, the CF foundation committee's consensus guidelines recommend a target 25OH Vit D level of 30 ng/mL or 75 nmol/L. It should also be noted that the recommended daily allowance of Vit D for non-CF individuals will not be adequate to maintain target levels in CF patients. Various possible replacement strategies have been suggested, but the optimal Vitamin D analog and dose (D2, D3, calcifediol, calcitriol, and UV therapy) remains to be determined.

### 14.2. Calcium Supplementation

Calcium is an important component of bone infrastructure and is essential to the maintenance of skeletal health. Schulze et al. showed that increased calcium absorption in young women with CF was associated with increased rates of bone calcium deposition [34]. Since there are no long-term randomized controlled trials in CF patients, appropriate supplemental doses are not defined and national dietary reference intakes should be followed. The CF foundation bone health consensus committee recommends supplementation with 1300 mg to 1500 mg of elemental calcium daily.

### 14.3. Vitamin K Supplementation

Vitamin K is necessary for posttranslational activation of osteocalcin, which in turn is important for bone formation and mineralization [42, 43]. Clinical practice guidelines recommend supplementation with 0.3 to 0.5 mg daily.

### 14.4. Gonadal Steroid Replacement Therapy

While adolescents with CF have lower sex hormone levels as compared to healthy age matched controls, most have normal values when adjusted for Tanner stage [48, 49]. The risk/benefit ratio of sex steroid replacement has not been adequately studied. Gonadal hormone replacement is controversial and should be limited only to those patients who have persistently low levels [13, 71]. Delayed puberty should be addressed by an experienced endocrinologist.

### 14.5. Antiresorptive Agents

Bisphosphonates are a class of drugs that chemically bind to calcium hydroxyl-apatite in bone and inhibit bone resorption through their inhibitory action on osteoclasts function and survival. Observational and randomized controlled trials (RCTs) on adults with CF have shown significant improvement in BMD with IV pamidronate and IV zoledronic acid as well as PO alendronate, but trials of these drugs in children with CF have not been performed [23–25, 65, 72, 73]. Oral agents are currently considered the first-line therapy for CF-related bone disease. The IV agents are frequently associated with side effects like fever, severe body aches, and bone pains [25, 65, 72], but concomitant use of steroids, acetaminophen, and/or nonsteroidals may help reduce this pain and fever syndrome. 

The current guidelines strongly recommend consideration of bisphosphonate therapy in all CF patients with T/Z-scores less than −2.0 and also in patients with T/Z score between −1.0 and −2.0, with a history of fragility fractures, those awaiting lung transplant, or who experience a BMD loss of >3%–5% per year. It is essential that when a bisphosphonate is used that the Vitamin D and calcium are adequately replaced prior to and during therapy.

### 14.6. Recombinant Human Growth Hormone

Children with CF are noted to have poor linear growth and inadequate weight gain as well as low levels of IGF-1. The use of growth hormone in children with CF has been shown to have a beneficial effect on linear growth and weight gain. There is evidence to suggest that treatment with recombinant human growth hormone results in improvement in clinical status with decreased hospitalizations and courses of intravenous antibiotics, improvement in exercise tolerance, and bone accumulation [74–79]. Human recombinant growth hormone has not been studied as a treatment for low BMD in adults with cystic fibrosis [74, 75, 77, 78, 80].

### 14.7. Teriparatide

Human parathyroid hormone has been shown to have both anabolic and catabolic effects on bone, depending on the dose and duration of use. While continuous exposure to high doses leads to bone resorption, daily low dose injections may actually increase osteoblast formation and bone growth [81–84]. Teriparatide (PTH 1–34) is the only anabolic agent available in the US for use in patients with advanced osteoporosis at high risk for fractures. Recombinant human (PTH 1–84) is available in Europe. It is contraindicated in children with open epiphyses but holds promise for CF adults with severe osteoporosis or prior history of fractures. There are currently no published studies of teriparatide in CF; however, this maybe a therapeutic option that should be considered and studied in the future for the adult population.

### 14.8. Newer Agents

Over the past few decades, researchers have made significant strides in the field of osteoporosis, which has led to an improved understanding of the regulation of bone remodeling. Exciting new treatment strategies have emerged and may potentially broaden our options for treatment of CF-related bone disease. Several of the therapies discussed are not clinically available currently but are under investigation and show promise to improve the bone health of the CF patients in the future.

#### 14.8.1. Antiresorptive Agents

Denosumab, a human mono-clonal antibody to RANKL (receptor activator of nuclear factor kappaB ligand), was recently approved by FDA for treatment of osteoporosis in postmenopausal women. Denosumab inhibits the maturation of osteoclasts by binding to RANKL [85]. The inhibition of osteoclast formation, function, and survival are responsible for decrease in osteoclast-mediated bone resorption. The improvement in lumbar and hip BMD seen with denosumab is at least comparable to bisphosphonates, if not superior. Denosumab also improves distal radius (cortical) bone density, an effect not seen with bisphosphonates [85]. Side effects noted with bisphosphonates such as delayed fracture healing, osteonecrosis of jaw, and femoral shaft fractures have not been observed in denosumab-treated individuals to date but further trials would be needed to assess the long-term effects of this medication [85]. The beneficial effect on both trabecular as well as cortical bone, combined with fewer side effects and the prospect of improved patient compliance due to twice-yearly dosing makes this an attractive consideration in CF-related bone disease. 

Cathepsins are a family of proteases with collagen as their main target. Cathepsin K (CAT k) is unique to osteoclasts and plays a role in degradation of bone matrix proteins, including collagen. CATk inhibitors have an antiresorptive effect, with lesser inhibition of bone formation than bisphosphonates. CATk inhibitors have been shown in animal studies to increase cortical thickness and periosteal bone formation and are currently undergoing phase 3 trials. They may have the potential to improve long bone strength and prevent nonvertebral fractures [86, 87].

#### 14.8.2. Anabolic Agents

**PTH ligands and PTHrP**

Antiresorptive agents can only increase bone mass but anabolic agents can also improve bone quality and strength, in addition to increasing bone mass. Currently, PTH [1–34] is the only approved anabolic agent in the US, and its duration of use is limited for 2 years. There is thus an unmet need for development of more anabolic agents.

Researchers have looked at various PTH ligands (PTH 1–31, PTH 1–28), which appear to have a more potent anabolic effect than the hormone itself (PTH 1–34) [88, 89]. Parathyroid hormone-related protein (PTHrP) is a hormone produced by mature osteoblasts and is structurally similar to PTH. Unlike PTH, which has both anabolic and catabolic effects on bone, PTHrP appears to be purely anabolic when administered intermittently. Studies in animals have shown an increase in trabecular bone volume, osteoblast number, bone mineralization rates, biomechanical strength, and BMD [90]. Human studies show increase in BMD similar to that with PTH, but without significant hypercalcemia or other adverse effects as observed with PTH. There is no activation of bone resorption at therapeutically effective doses [91–93].

#### 14.8.3. Calcilytic Agents

Allosteric modulations of the G-protein coupled calcium-sensing receptor in the parathyroid gland can affect PTH release. Positive allosteric modulators decrease PTH production, while negative modulators increase it. Calcilytics or calcium sensing channel antagonists are negative modulators, which when administered orally, can cause a pulsatile increase in PTH production. Animal studies look promising; unfortunately, a human trial of ronacaleret was discontinued due to a poor effect on BMD. Another agent is currently in phase 2 trials [94].

#### 14.8.4. Modulation of the Wnt Signaling Pathway

**Antisclerostin Antibody**

A large family of extracellular glycoproteins, called Wnt proteins, are key regulators of bone remodeling and other cellular processes [95–97]. Sclerosteosis, an autosomal recessive disorder characterized by increased bone mass, mainly in the skull and in long bones, results from a mutation in the *SOST* gene, which codes for sclerostin [98]. A deficiency of sclerostin results in increased Wnt signaling and high bone mass. Transgenic mice that overexpress sclerostin are seen to have low bone mass and increased susceptibility to fractures [99] while mice deficient in sclerostin show increased bone density [100]. Treatment with sclerostin antibody has been associated with an increase in bone mass and strength in various animal models [101–103]. In a phase I trial in 72 healthy men and postmenopausal women, a single subcutaneous dose of sclerostin antibody was associated with a statistically significant increase in the levels of bone formation markers propeptide of type I procollagen (P1NP), osteocalcin and bone-specific alkaline phosphatase (BSAP), a dose-dependent decrease in bone resorption marker C-telopeptide, and a 5.3% increase in lumbar spine BMD [104]. Antisclerostin antibody seems promising as a potentially effective anabolic agent for the treatment of low bone mass in individuals with CF.

**Anti-Dkk1 Antibody**

Dickoppf1 (DKK1) is a naturally occurring Wnt pathway antagonist which works by preventing the interaction between two key Wnt pathway coreceptors, LRP5/6 and the frizzled Wnt pathway receptor, which results in inhibition of Wnt signal transduction and impaired bone formation [105]. DKK1 inhibition increases trabecular bone volume and bone formation in rats [106, 107]. Antibodies to dickkopf-1 could be used as an anabolic agent for the treatment of patients with low bone mass.

**Activin Inhibitors**

Activin is a member of the bone morphogenic protein (BMP)/transforming growth factor (TGF)*β* superfamily of polypeptides and stimulates the release of FSH by the pituitary gland [108]. In bone, activin binds to activin receptor IIA, increases osteoclastogenesis, and is a negative regulator of bone mass. Its effects on bone formation are more complex and unclear [109].

ACE-011, a protein formed by fusing soluble activin receptor type II to IgG-Fc, was shown to decreases bone resorption in ovariectomized mice and enhance bone formation in intact animals [110]. Data in cynomolgus monkeys shows a significantly higher BMD and trabecular bone volume after injection of ACE-011 [111]. In phase I trials, the administration of a single dose to 48 postmenopausal women resulted in an increase in levels of bone-specific alkaline phosphatase and a decrease in C-telopeptides [112]. Activin inhibitors hold promise as new anabolic therapy for patients with low bone mass and increased susceptibility for fractures.

## 15. Conclusion

CF-related bone disease is a common complication in the aging CF population. It is multifactorial in origin, affects young individuals, and is associated with significant morbidity. Despite increasing research in the field, numerous questions about pathogenesis and appropriate therapy still remain. A multisystemic, multidisciplinary approach is required to tackle bone disease in the CF population. Various therapies are currently available for treatment of postmenopausal osteoporosis but these have not been studied in individuals with CF. Clinical trial data is especially limited in the pediatric age group. The new Cystic Fibrosis Foundation guidelines for the treatment of bone disease are expected to be published in the next year. Exciting new therapies are currently under development, and investigation and hold promise for reducing the burden of secondary bone disease in individuals with CF.

## Figures and Tables

**Table 1:** Causes of decreased bone density in cystic fibrosis.

(i) Pancreatic insufficiency	
(ii) Malnutrition and poor growth	
(iii) Vitamin D, vitamin K, and calcium insufficiency	
(iv) CF-related diabetes	
(v) Glucocorticoids	
(vi) Sex steroid deficiency and delayed puberty	
(vii) Chronic inflammation	
(viii) Tobacco, alcohol, and caffeine use	
(ix) Moderate to severe lung disease	
(x) Lack of exercise, especially weight bearing	
(xi) Organ transplant and immunosuppressive therapy	
(xii) Medications (Depo-Provera, Megestrol Acetate, and Aluminium containing-antiacids)	
(xiii) Possible role of CFTR.	

**Table 2:** Cystic fibrosis foundation guidelines for treatment of osteoporosis.

	**General Recommendations**		

Vitamin D goal	25OH Vit D level >75 nmol/L or 30 ng/mL		

Calcium	1300–1500mg/day		

Vitamin K	0.3–0.5 mg/day		

Target BMI	>50th percentile		
	BMI adult females 23		
	BMI adult males 22		

Exercise	Encourage outdoor and weight bearing exercise		

Infections	Aggressive treatment of pulmonary infections		

	**Specific Recommendations by T/Z Score**		

	**T/Z score ≤ −2**	**−1≤ T/Z score ≤ −2**	**T/Z scores ≥ −1.0**

Repeat DEXA	Annual	Repeated every 2–4 years	Repeated every 5 years

Steroid	Minimize steroid doses		No recommendation

Endocrine	Recognize and treat CF related diabetes, delayed puberty, hypogonadism, and consider endocrine consult		No recommendation

Bisphosphonate	Start bisphosphonate	If fragility fracture, patient awaiting transplant or accelerated BMD loss >3-5% per year start bisphosphonate	No recommendation
